# Sex-biased expression between guppies varying in the presence of ornamental coloration

**DOI:** 10.7717/peerj.5782

**Published:** 2018-10-11

**Authors:** Cynthia Dick, David N. Reznick, Cheryl Y. Hayashi

**Affiliations:** 1 Department of Evolution, Ecology, and Organismal Biology, University of California, Riverside, Riverside, CA, United States of America; 2 Division of Invertebrate Zoology and Sackler Institute for Comparative Genomics, American Museum of Natural History, New York, NY, United States of America

**Keywords:** Transcriptome, Pigmentation, Sexual dimorphism, Color genes, Guppy

## Abstract

Sex-biased gene expression provides a means to achieve sexual dimorphism across a genome largely shared by both sexes. Trinidadian guppies are ideal to examine questions of sex-bias as they exhibit sexual dimorphism in ornamental coloration with male only expression. Here we use RNA-sequencing to quantify whole transcriptome gene expression differences, with a focus on differential expression of color genes between the sexes. We determine whether males express genes positively correlated with coloration at higher levels than females. We find that all the differentially expressed color genes were more highly expressed by males. Males also expressed all known black melanin synthesis genes at higher levels than females, regardless of whether the gene was significantly differentially expressed in the analysis. These differences correlated with the visual color differences between sexes at the stage sampled, as all males had ornamental black coloration apparent. We propose that sexual dimorphism in ornamental coloration is caused by male-biased expression of color genes.

## Introduction

Phenotypic divergence in morphological traits can lead to the evolution of dimorphism within species. Dimorphism has occurred between morphs or subspecies such as freshwater and marine stickleback (Colosimo et al., 2005), cave and surface Mexican tetra (Protas et al., 2006), or light and dark colored mice (Hoekstra, 2006). In addition, substantial phenotypic differences can occur between males and females of a species (Lande, 1980). This sexual dimorphism can result from factors such as natural selection, sexual selection, or sexual conflict. Sexual dimorphism can be caused by natural selection if traits are favored that increase survival or reproduction in one sex (Ellegren & Parsch, 2007). Sexual dimorphism via sexual selection could occur for traits directly involved in mating success and can include intersexual and intrasexual selection (Ellegren & Parsch, 2007). Sexual conflict may happen when a trait is beneficial to one sex and detrimental to the other (Lande, 1980; Pennell & Morrow, 2013).

The cause of sexual dimorphism demands an explanation because males and females share nearly all of their genome. In one case, genes coding for or regulating the trait could be genetically linked to a non-recombining region of the heterogametic sex chromosome (Basolo, 2006; Kirkpatrick & Hall, 2004). Alternatively, sex-biased expression can occur, where the gene(s) for the trait are expressed exclusively or more highly by one sex (Ellegren & Parsch, 2007). Sex-bias in gene expression, as opposed to sequence alterations, is the focus of this study since gene expression differences are thought to account for a majority of sexual dimorphism (Ellegren & Parsch, 2007; Mank, 2009). For example, a study on birds found that sexual selection had a greater impact on gene expression evolution than sequence evolution (Harrison et al., 2015).

A link between sexual dimorphism and sex-biased expression has been found in many species varying from highly sexually dimorphic birds to lowly sexually dimorphic alga (Lipinska et al., 2015; Pointer et al., 2013). Sex-biased expression of color genes was recently discovered in damselflies, although females of this species are the ones that exhibit color polymorphism and so there are greater numbers of female-biased genes across the transcriptome compared to male-biased genes (Chauhan, Wellenreuther & Hansson, 2016). Previous studies of sex-biased expression in guppies that included caudal peduncle tissue (located on the body) found 33 color genes with differential expression, 29 of which were male-biased (Sharma et al., 2014). These and other analyses of variation in sexually dimorphic traits require correlating the expression level of sex-biased genes with sex-specific phenotypic variation.

**Figure 1:**
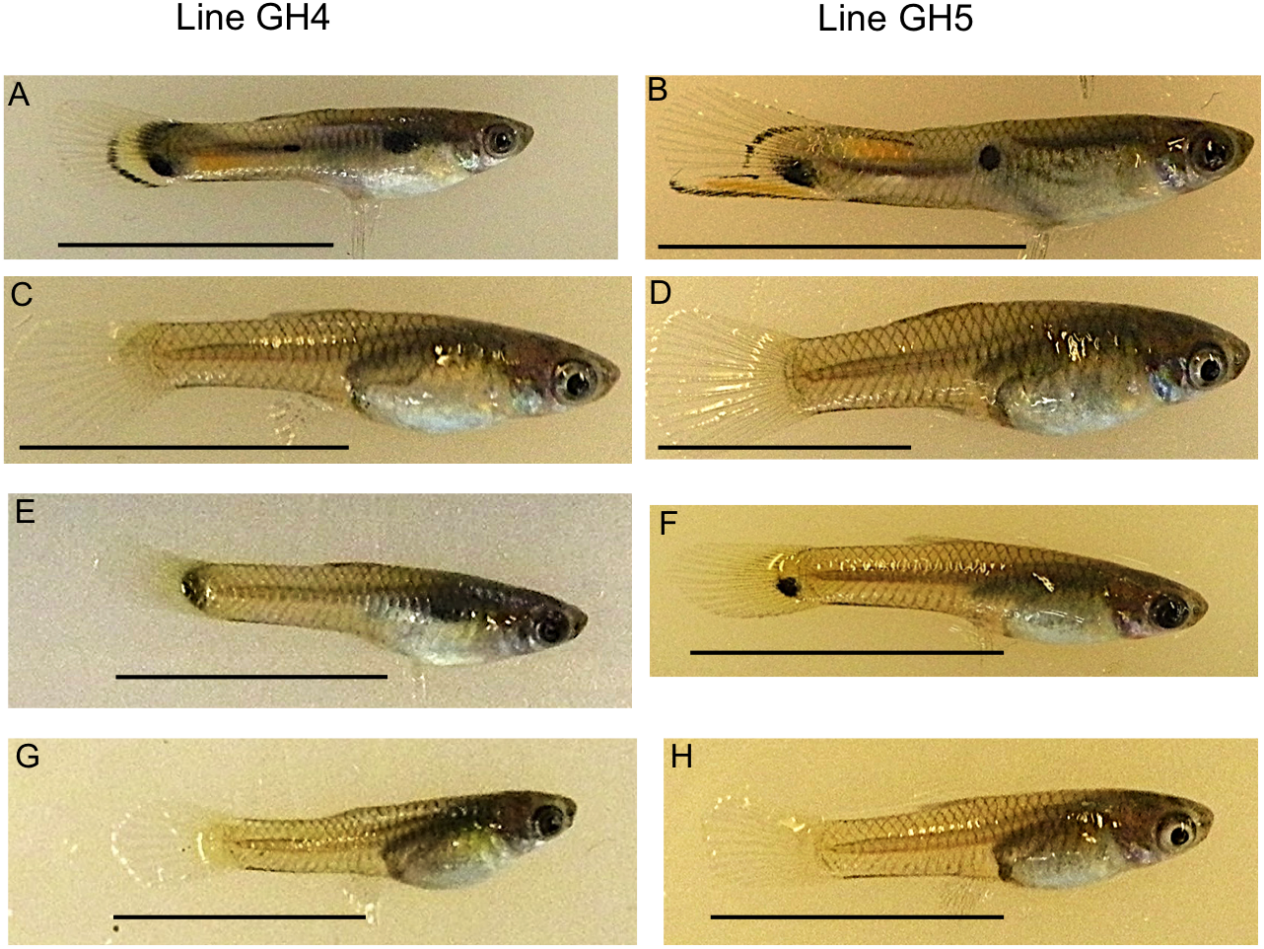
Example guppies from the two iso-male lines sampled. Adult males from these lines have ornamental coloration (A, B), while females (C, D) do not. Ten maturing caudal fins were sampled per sex and line for RNA-seq analysis. Photographs show an example male (E, F) and female (G, H) from each line. Scale bar 1 cm.

The Trinidadian guppy (*Poecilia reticulata*) provides an ideal system to examine sex-biased expression of male ornamental coloration, an evolutionarily relevant trait (Sharma et al., 2014). By comparing males and females, it is possible to learn about the identity and functions of genes that may be important in generating phenotypic variation. These genes can be used as candidates for future studies examining phenotypic color variation in other groups of guppies, such as guppies from different predator communities, which are known to have differences in coloration (Ender, 1980). Guppies have an XY sex determination system with males as the heterogametic sex (Winge, 1922). Both male and female guppies have a reticulate black camouflage and light yellow color to their bodies, but only males possess ornamental coloration (Fig. 1). The ornament gradually develops on the body and caudal fin as the male matures (Fig. 1). Ornamental coloration in guppies represents a trade-off between sexual and natural selection (Endler, 1980). Sexual selection favors bright coloration, while natural selection by diurnal, visually oriented fish predators favors duller coloration. Early studies determined that ornamental color pattern elements have a variety of linkage patterns (Haskins & Haskins, 1951; Winge, 1927). Many of these were Y-linked, although Y linkage can vary among geographic sites. For example, high predation fish have more Y-linked body coloration, while low predation fish have more autosomal and X linkage (Gordon, Lopez-Sepulcre & Reznick, 2011; Haskins et al., 1961). These results could be due to selection on standing genetic variation for non-Y-linked color or increased recombination rates between X and Y chromosomes in low predation fish (Gordon, Lopez-Sepulcre & Reznick, 2011). Since female choice for male coloration is more predominant in low predation environments, selection on gene dosage or selection for a genetic correlation between female preference and male coloration could favor X- or autosomal-linkage (Gordon, Lopez-Sepulcre & Reznick, 2011).

Although, sex-bias of male coloration has been previously examined in guppies, tissue from the body was used (Sharma et al., 2014). Since all guppies have camouflage body coloration, it is hard to disentangle genes involved in ornamental color variation from genes that create the camouflage pattern found in both males and females. To separate expression of sex-specific ornamental versus non-sex-specific coloration, tissue from the caudal fin is used here. The caudal fin is the only skin location where there are reliable sex-specific differences and a lack of camouflage coloration that would otherwise prevent the detection of sex-specific ornamental coloration. Caudal fin coloration is used in courtship displays (Farr, 1980) and is subject to both natural and sexual selection (Hughes et al., 2013; Olendorf et al., 2006). Although body coloration extends slightly into the base of the caudal fin of both sexes, females largely have clear caudal fins.

The goal of this study was to characterize sex-biased expression in guppy caudal fin color genes. Ornamental caudal fin coloration in the males includes black melanin, orange/yellow pteridines or carotenoids, and shimmering iridescence. The color genes that we examine regulate color in a positive way or synthesize the color. Therefore, we hypothesize that any sex-biased genes will have higher expression in males than females as only males express ornamental color. Since the fish were sampled at a stage when male melanin coloration was beginning to appear, we also specifically hypothesize that males will express melanin synthesis genes at consistently higher levels than females.

## Materials and Methods

### Sampling and RNA extractions

Guppies were collected from a high predation (HP) site in the Guanapo River in the Northern Range Mountains of Trinidad. Crosses were performed to generate two different iso-male lines. Each line was initiated from a single wild-caught male and female. Male and female F1 laboratory offspring were allowed to inbreed to generate the F2 generation, which was used for sampling. The lines had slightly different final male color patterns (Fig. 1). However, our question addressed whether color genes were associated with the presence of any ornamental coloration, rather than associating genes with specific patterns. Fish were bred for two generations in the laboratory at University of California, Riverside (UCR; IACUC AUP approval: A-20140003). Male caudal fins were sampled at a time when their anal fins were morphing into the gonopodium, which also correlated with early development of caudal fin coloration. This time was chosen because we found in an earlier study that males at this stage expressed the highest number of detectable color genes and it was simpler to obtain fish approximately the same age while their anal fins were still in the process of morphing (Dick et al., in press). Female fins were also collected, although females lack an independent marker of maturation. Therefore, females were taken from the same litter as the males so were the same age and genetic background. Fish were anaesthetized in MS-222 and 10 caudal fins from sibling males or females were removed, combined into a single sample per sex, and frozen in liquid nitrogen. Ten tails per sample were collected as prior research indicated 10 caudal fins were needed for successful RNA extraction. Each tail was approximately 0.2 cm^2^. Due to Y-linkage of male color patterns, all brothers within a line had nearly identical patterns. This sampling was done for each line for a total of four samples and two biological replicates per sex. Although it is preferable to include more replicates, funding and vivarium space were limited. The differential expression analysis program used (see below) can accommodate two biological replicates and maintain the false positive rate below threshold values (Robles et al., 2012). Therefore, it is more likely that significantly differentially expressed (DE) genes would be overlooked due to reduced power compared to mistakenly calling a truly non-DE gene as DE. After caudal fin collection, fish were sacrificed in a lethal dose of MS-222 with no recovery in between. Samples were stored at −80 °C until RNA extraction. The two male samples are the same as obtained during a developmental series study (Dick et al., in press). These two samples were taken at an early developing color stage (called stage 2), where usually just black melanin was present.

Caudal fins were homogenized in Trizol (Invitrogen, Carlsbad, CA, USA) using a Tissue Tearor (BioSpec Products, Bartlesville, OK, USA). Total RNA was purified using a Qiagen RNeasy Mini Kit (Valencia, CA, USA) and then treated with TURBO DNase (Ambion, Carlsbad, CA, USA). RNA was quantified with a Qubit 2.0 Fluorometer (Invitrogen) and integrity was measured with an Agilent Bioanalyzer (Santa Clara, CA, USA). All samples had RNA Integrity values ≥ 7.7.

### Illumina sequencing

RNA-seq libraries were prepared by the University of California, San Diego Institute for Genomic Medicine using the Illumina TruSeq unstranded RNA Library Preparation kit v2 (San Diego, CA, USA). The manufacturer’s recommendations were followed and each sample received a unique barcode. Samples were pooled into equimolar amounts on three separate flowcells. The GH4 lines were sequenced on one flowcell containing nine unrelated samples, while the two GH5 samples were sequenced on two other separate flowcells containing four samples each from an unrelated experiment. Samples were sequenced on an Illumina HiSeq2500 at UCR using 100 bp single-end sequencing. Each sample yielded between 8.5–33.9 million reads (Table 1). Sequence reads have been deposited in NCBI’s Sequence Read Archive (Accession: SRP111128).

**Table 1:** Number of RNA sequencing reads obtained for each sample. Reads were mapped to the annotated guppy genome (NCBI GCF_000633615.1).

**Line**	**Replicate**	**Sex**	**Raw reads**	**Cleaned reads**	**Mapped reads (%)**
GH4	1	M	8,527,408	6,397,511	5,835,774 (91.2)
GH5	2	M	33,937,307	26,510,922	24,218,195 (91.5)
GH4	1	F	10,227,445	7,896,411	7,174,677 (90.9)
GH5	2	F	29,135,360	18,989,337	18,986,132 (92.3)

### Quality control, alignment, read counting and differential expression

RNA-seq reads were cleaned using the fastq_quality_filter tool of the FASTX-Toolkit (Hannon Lab, Cold Spring Harbor Laboratory, NY). Reads were required to have a Phred +33 quality score of at least 20 in 100% of bases. Residual adapter sequences were then trimmed using Trimmomatic 0.20 (Bolger, Lohse & Usadel, 2014). The first 12 bps of each read were removed after not getting passing scores in the FASTQC report (Babraham Institute, Cambridge, UK). Reads with a minimum length less than 50 bps were discarded. This left 6.4–26.5 million reads per sample (Table 1).

Cleaned reads were aligned to the annotated guppy genome (Kunstner et al., 2016; NCBI accession GCF_000633615.1) using TopHat2 (Trapnell, Pachter & Salzberg, 2009). Default options were used except the number of threads was four and the minimum intron size was 50 bps. The output BAM files from TopHat2 were sorted by sequence name and converted to SAM files. Read counts for each gene were obtained using ht-seqcount in the union mode (Anders, Pyl & Huber, 2015).

Differential expression analysis was performed using DESeq2 (Anders & Huber, 2010) in R version 3.1 ( R Core Team, 2014). Although there was variation in the total number of reads mapped per sample, DESeq2 is able to handle varying read counts (Anders & Huber, 2010). Briefly, DESeq2 calculates a size factor, based on sampling depth, for each library and then scales gene counts by this coverage value. It does this prior to performing differential expression estimates. Samples were grouped according to sex so that the two different lines were considered biological replicates. Genes considered for differential expression were required to have at least one count per million mapped reads (CPM) for at least two samples. A contrast was generated between males and females and differential expression was tested using a false-discovery rate cutoff of 0.05 (Benjamini & Hochberg, 1995). There was no threshold in log_2_ fold changes to call a gene significant, although all DE genes had log_2_ fold-change values ≥1.1, corresponding to over a two-fold change in expression level.

Genes known to be involved in coloration were the focus and were obtained from multiple citations (Table S1). Special attention was also paid to genes listed by Braasch et al. (2009) under the functions “pteridine synthesis” or “components of melanosomes” and by Walsh et al. (2012) under the function “carotenoid related”. Although three of the carotenoid genes have functions that may preclude expression in the caudal fin (*bcmo1*, *scarb1*, and *scarb2*), they were retained in the analysis. Individual gene expression levels were calculated from counts per million mapped reads.

## Results

Males and females had similar percentages of reads mapping to the guppy genome (Table 1). The guppy genome contains 26,071 loci and 18,568 met the cut-off imposed for differential expression estimates of at least one count per million in at least two samples. Almost all of the color genes tested had annotations in the guppy genome. Since the genome was assembled from a female guppy, these color genes must be X- or autosomal-linked. Of the 106 genes with at least one function in coloration annotated in the guppy genome, 102 met the cut-off imposed as above.

**Figure 2:**
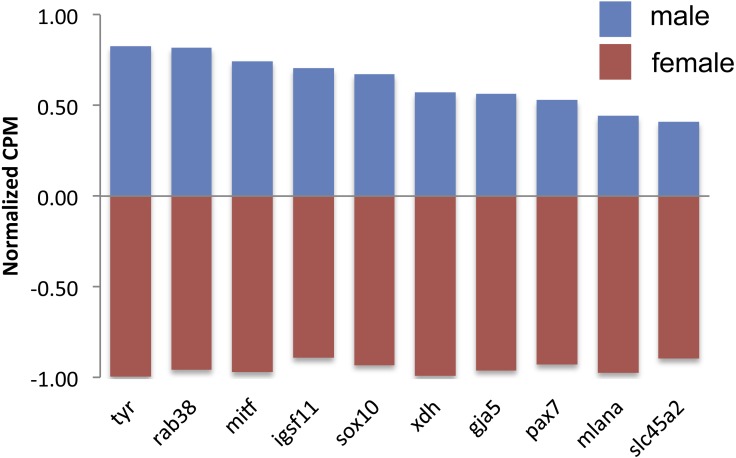
Expression of differentially expressed color genes by male (blue) and female (red) guppies. Normalization of counts per million mapped reads (CPM) was performed so all genes could be graphed on the same axis. To do so, feature scaling was applied to each sample and then the scaled values were averaged within sex to yield a single male and single female normalized value bounded by −1 and +1. Feature scaling for each gene was calculated as: *x* + ((sampleCPM–minimumCPM)* (*y* − *x*)/(maximumCPM–minimumCPM)), where *x* =  − 1 and *y* =  + 1. If the biological replicates within sexes tended to agree, then one sex would have a positive value and the other sex would have a negative value.

There were 124 differentially expressed (DE) genes between males and females, with males having higher expression of 123 of those genes. Of these genes, ten color genes were DE between sexes, with all ten more highly expressed in males (Fig. 2, Table S2). Six of these 10 DE color genes are involved in the eventual formation of black melanin pigmentation (Table 2), which correlates with the visual presence of black coloration at the stage sampled. The other four genes had more general, unknown, or pteridine synthesis functions.

**Figure 3:**
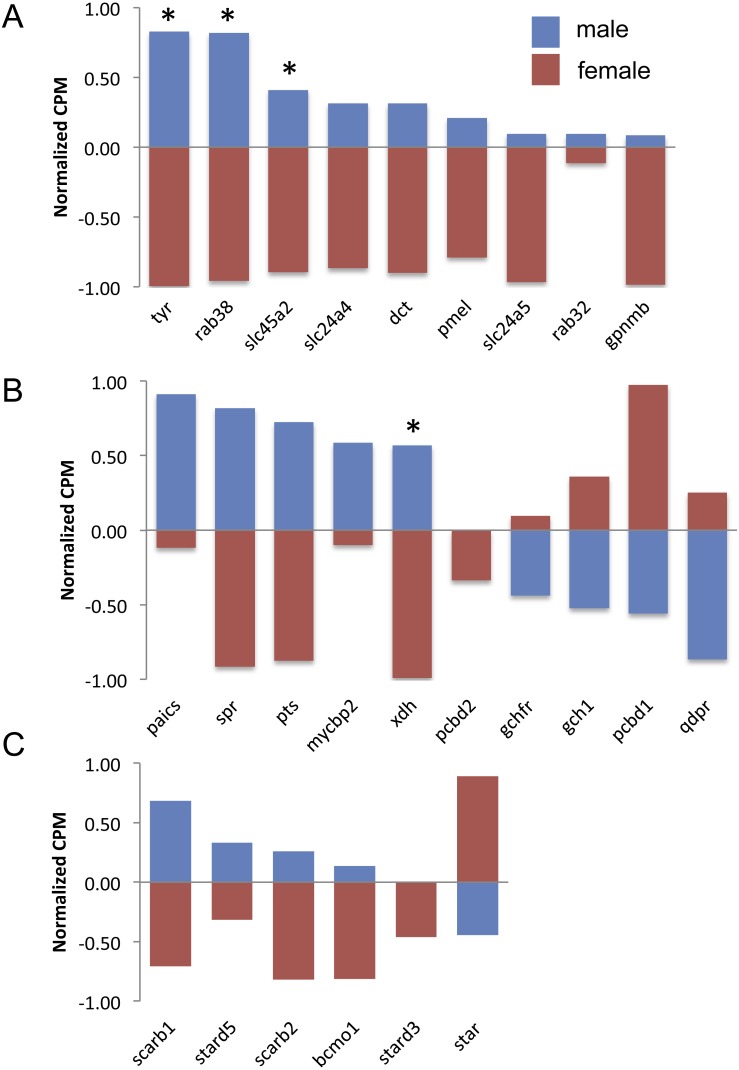
Expression of melanin synthesis (A), pteridine synthesis (B) and carotenoid (C) genes by male (blue) and female (red) guppies. Normalization and feature scaling was performed as in Fig. 2. Asterisks above bars indicate genes that were differentially expressed between sexes.

There are nine genes exclusively involved in melanin synthesis in the guppy genome. Three of them were DE in the analysis, but all of them consistently had higher expression in males (Fig. 3A), which also correlates with the presence of black in male caudal fins only. There was less of a clear trend to whether males or females more highly expressed orange/yellow pteridine synthesis genes and only one gene was DE (Fig. 3B). Orange/yellow carotenoid genes usually had higher expression in males, but none were DE (Fig. 3C).

**Table 2:** Identification, function, and final color type expressed of color genes more highly differentially expressed by males.

**Gene name**	**Function**	**Final color type expressed**
*gja5*	Pattern formation	Melanin/pteridines
*igsf11*	Melanin pattern formation	Melanin
*mitf*	Melanophore development	Melanin
*mlana*	Melanogenesis regulation	Melanin
*pax7*	Xanthophore development	Pteridines or carotenoids
*rab38*	Melanosome components	Melanin
*slc45a2*	Melanosome components	Melanin
*sox10*	Chromatophore development	Any
*tyr*	Melanosome components	Melanin
*xdh*	Pteridine synthesis	Pteridines

## Discussion

Our hypothesis that males with ornamental pigmentation would have increased expression of color genes was supported. Specifically, males had significantly more highly expressed color genes than females. Studies in seven *Drosophila* species and one bulb mite species have also shown that the direction of sex-bias usually favors more male-biased genes (Stuglik et al., 2014; Zhang et al., 2007). However, we found a much greater disparity in the number of genes significantly more highly expressed by males. It is unclear what could cause this large disparity, although caudal fins are important for swimming performance. Males usually have longer caudal fins that cause altered swimming performance (Karino, Orita & Sato, 2006) so may have increased expression levels of genes with functions that contribute to swimming performance. Sexual dimorphism in ornamental coloration is at least partly caused by male-biased expression of color genes. We predict that these differences in color gene expression promote the formation of male ornamental color patterns and are advantageous during courtship of females. However, there may still be trade-offs between predation risk and sexual selection (Endler, 1980).

There were 10 DE color genes (*gja5*, *igsf11, mitf*, *mlana*, *pax7*, *rab38*, *slc45a2*, *sox10*, *tyr*, *xdh*) between males and females, with all 10 more highly expressed by males. Two of the most male-biased color genes found in Sharma et al. (2014) (*tyr*, *xdh*) were also found to be DE in this study. Although the *gja5* gene is uncategorized by Braasch et al. (2009), another study found that zebrafish with *gja5* mutations had a reduction of melanophores (Watanabe et al., 2006). *Igsf11* is involved in adult melanin pattern formation and zebrafish mutant in this gene have defects in the survival and migration of melanophores (Eom et al., 2012). *Mitf* is involved in melanophore development and positively regulates *mlana* (Du et al., 2003), which can both regulate a melanin synthesis gene. *Pax7* is expressed in early xanthophore cells and mutants have reduced yellow pigmentation (Minchin & Hughes, 2008). *Rab38* targets a melanin synthesis enzyme to the melanophores (Montoliu, Oetting & Bennet, 2011). *Slc45a2* and *tyr* have functions in producing melanin and mice mutant in these genes have reduced or absent melanin pigmentation (Montoliu, Oetting & Bennet, 2011). *Sox10* is involved in differentiation from the neural crest cell and mutants have pigmentation defects in addition to defects in the peripheral nervous system (Dutton et al., 2001). *Xdh* plays an ultimate role in the synthesis of pteridines, specifically yellow sepiapterins (Braasch, Schartl & Volff, 2007).

Most of these sex-biased DE genes have general functions in the regulation of melanophores, xanthophores, or neural crest cells (Braasch et al., 2009). These genes may affect more than color pattern formation, in contrast to genes whose only function is to synthesize melanin or pteridines. Future studies could confirm the pleiotropic effects of these genes. Since all females still have melanin camouflage body coloration, regional and tissue-specific differences in gene regulation are clearly important in generating the complex trait that is guppy ornamental coloration. We expect trait variation among color polymorphic males is at least partially explained by regulatory changes in transcription regulators and developmental genes since genes with these functions were DE.

Our hypothesis that melanin synthesis genes would be more highly expressed in males was supported. More than half of the differentially expressed genes (6/10) had functions related to melanin and all melanin synthesis genes, regardless of differential expression status, were more highly expressed in males. This agrees with our hypothesis that melanin genes would exhibit more sex-bias than pteridines or carotenoids because we sampled the fish at a stage when the melanin was visually appearing and melanin synthesis gene expression is known to increase (Dick et al., in press).

The differential expression of color genes that we identified could have resulted if there was sexual conflict over the expression of these genes between male and female guppies. A recent study in guppies examined X–Y divergence at high and low predation locations in three independent drainages (Wright et al., 2017). Males in low predation locations are known to have increased ornamental coloration compared to high predation localities because female preference for brightly colored males is not offset by brightly colored males being more susceptible to predation (Endler, 1980). The authors repeatedly and independently found expansion of the non-recombining region in low predation locations with resulting X–Y divergence. They proposed that sexual antagonism over aspects of coloration linked to the sex chromosomes could have generated the suppressed recombination (Wright et al., 2017). Another study in African cichlids found sexual antagonism over the orange-blotch pattern thought to be more beneficial to female camouflage coloration. The orange-blotch pattern was associated with increased expression of the gene *pax7*, which was in turn linked to the female sex-determining region (Roberts, Ser & Kocher, 2009). Our study identified differential expression of *pax7*, although here males had the higher expression.

All genes with male-biased expression that we identified here are candidates for future studies of sexual conflict. For example, these genes can be used to test correlations between gene expression level and sex-specific fitness. In addition, studies utilizing fish from low predation locations could be used to determine the impacts of predator community on gene expression of color genes between the sexes. This would be of interest since the fish used in the present study are from a high predation site only.

## Conclusions

We find that color gene expression positively correlates with the presence of guppy ornamental coloration. Genes exhibiting significant differential expression were always more highly expressed in males compared to females. This matched our hypothesis given that the genes of interest are known to be positively associated with color. In addition, all melanin synthesis genes had higher expression in males, regardless of differential expression status. Additional studies examining genes in variably colored guppies at the developmental stage sampled here are likely to find that melanin synthesis genes are important candidates. Adding more biological replicates per sex will increase the statistical power for detecting DE and hence may identify additional DE genes. In addition, future studies could examine the set of differentially expressed genes across the entire transcriptome to determine the functions of non-color genes with sex-bias.

##  Supplemental Information

10.7717/peerj.5782/supp-1 Table S1 Color genes annotated in male and female Trinidadian guppies Expression estimates for each sample are given in counts per million mapped reads (CPM). Positive log2 fold-changes indicate higher expression in males, while negative numbers indicated higher expression in females. Linkage groups in guppies largely correspond to chromosomes, with LG12 being the X chromosome. Listed also is the gene function from the reference where the identity of the color gene was described.

10.7717/peerj.5782/supp-2 Table S2 Genes found to be significantly differentially expressed (DE) between male and female Trinidadian guppies Positive log2FC indicates higher expression in males and negative values indicate higher expression in females. Genes known to be involved in coloration are shown in red. LOC genes have unknown annotations. Linkage groups in guppies largely correspond to chromosomes, with LG12 being the X chromosome.

## Additional Information and Declarations

### DNA Deposition

The following information was supplied regarding the deposition of DNA sequences: Raw RNA-seq reads have been uploaded to NCBI SRA (Accession: SRP111128).

### Data Availability

The following information was supplied regarding data availability: The raw data are provided as Supplemental Files.
